# Exploratory Identification of Multidimensional COPD Clusters Using Unsupervised Analysis

**DOI:** 10.3390/medicina62091656

**Published:** 2026-08-29

**Authors:** Andrea Portacci, Mariafrancesca Grimaldi, Maria Rosaria Vulpi, Carla Santomasi, Fabrizio Diaferia, Alessandro Capuano, Giovanni Sanasi, Marianna Cicchetti, Eustachio Ricciardi, Alfredo Vozza, Giulia Amoroso, Alessio Marinelli, Vitaliano Nicola Quaranta, Silvano Dragonieri, Giovanna Elisiana Carpagnano

**Affiliations:** 1 Institute of Respiratory Disease, Department of Translational Biomedicine and Neuroscience, University “Aldo Moro”, 70121 Bari, Italy; a.portacci01@gmail.com (A.P.); mariafrancescagrimaldi@gmail.com (M.G.); mariarosaria.vulpi@gmail.com (M.R.V.); carlasantomasi@gmail.com (C.S.); fabrizio.diaferia@gmail.com (F.D.); alecapuano92@gmail.com (A.C.); g.sanasi@studenti.uniba.it (G.S.); mariannacic@gmail.com (M.C.); eus.ricciardi@gmail.com (E.R.); giulia.amoroso@uniba.it (G.A.); alessio.marinelli@uniba.it (A.M.); vitalianonicola.40@gmail.com (V.N.Q.); elisiana.carpagnano@uniba.it (G.E.C.); 2 Department of Precision and Regenerative Medicine and Ionian Area, University of Bari “Aldo Moro”, 70121 Bari, Italy; alfredovozza@live.it

**Keywords:** COPD, cluster analysis, phenotypes, blood eosinophils, emphysema

## Abstract

*Background and Objectives*: COPD is a heterogeneous disease in which conventional clinical classifications may not fully capture the complexity of patient profiles. This exploratory study aimed to examine whether multidimensional COPD phenotypes could be identified using unsupervised cluster analysis integrating clinical, functional, radiological and laboratory features. *Materials and Methods*: We enrolled 161 patients with confirmed COPD evaluated between January 2020 and January 2024. Demographic, clinical, functional, radiological and laboratory findings were collected. Mixed-type data were analyzed using Gower distance and Partitioning Around Medoids (PAM) clustering. The optimal solution was selected by average silhouette width; stability was assessed by 1000 bootstrap resamples and sensitivity analyses. *Results*: The two-cluster solution had the highest silhouette width (0.184), although separation was modest. Cluster 1 (*n* = 78) was characterized by greater symptom and exacerbation burden, worse lung function, greater static hyperinflation, shorter 6-min walking distance and more frequent emphysema than cluster 2 (*n* = 83). Bootstrap resampling indicated internal stability, although concordance with the primary partition varied across sensitivity analyses. After correction for multiple post hoc comparisons, only LAMA/LABA/ICS use differed between clusters, whereas demographic characteristics, comorbidity burden and blood eosinophil levels were comparable. *Conclusions*: These exploratory findings suggest multidimensional assessment may complement conventional classifications, but external and longitudinal validation is needed before clinical implementation.

## 1. Introduction

The GOLD report 2025 defines Chronic Obstructive Pulmonary Disease (COPD) as a heterogeneous condition characterized by chronic respiratory symptoms due to airway or parenchymal abnormalities which cause persistent and often progressive airflow obstruction [1]. This heterogeneity is reflected in the wide variability of clinical presentation, structural damage, inflammatory profiles and disease progression observed among patients [2]. Recent advances in our understanding of COPD pathophysiologic mechanisms led to the recognition of specific disease-related treatable traits which could be targeted for more tailored therapeutic strategies [3]. Functional impairment, radiological patterns, systemic inflammation and comorbidity burden frequently coexist in different combinations, suggesting the presence of biologically and clinically distinct subgroups. Among the proposed treatable traits, blood eosinophil count (BEC) has emerged as a relevant biomarker, particularly in predicting response to inhaled corticosteroids (ICS) and to biologic therapies targeting type 2 inflammation [4,5]. However, eosinophilia represents only one dimension of COPD heterogeneity. Even within patients with similar BEC values, clinical outcomes and structural characteristics may differ substantially, suggesting that eosinophilic status alone is insufficient to define disease phenotype [6,7].

Previous cluster analyses have shown that COPD heterogeneity extends beyond functional severity, although the resulting phenotypes have varied according to the study population and the variables included. In a French multicentre cohort, Burgel et al. used principal component analysis followed by cluster analysis to identify four clinical phenotypes differing in age, symptoms, lung function, comorbidities and predicted mortality [8,9]. Within the Evaluation of COPD Longitudinally to Identify Predictive Surrogate End-points (ECLIPSE) cohort, marked clinical and radiological variability was observed among patients with similar degrees of airflow limitation [2], while a subsequent cluster analysis of 2164 patients identified five subgroups with different inflammatory profiles and three-year outcomes [10]. Other studies, including Phenotype and Course of Chronic Obstructive Pulmonary Disease (PAC-COPD) and COPDGene, generated different clustering solutions in hospital-based populations and extensively characterized research cohorts [11,12]. More recently, Zucchi et al. identified an eosinophil-rich cluster when blood eosinophil count was included among the clustering variables [13].

However, the variability of the reported clustering solutions indicates that the resulting profiles are strongly influenced by the population studied and the variables selected and their applicability to routine clinical settings remains uncertain. Moreover, qualitative HRCT abnormalities have been less extensively integrated with clinical, functional and inflammatory features, while studies specifically addressing eosinophilic heterogeneity have generally included BEC among the clustering variables. Considering these aspects, it is still unclear whether BEC can characterize multidimensional COPD profiles derived independently of eosinophil values. To address these gaps, we applied unsupervised cluster analysis to a real-life outpatient COPD population by integrating clinical, functional, radiological and selected laboratory features, while excluding BEC from cluster derivation and subsequently examining its distribution across the resulting data-driven profiles.

## 2. Materials and Methods

### 2.1. Study Population

We performed a real-life, retrospective, observational study enrolling patients with a confirmed diagnosis of COPD following GOLD recommendations from January 2020 to January 2024. COPD diagnosis required persistent airflow obstruction, defined as a post-bronchodilator FEV_1_/FVC ratio < 0.70.

Data retrieved from the electronic medical records of patients visited in our outpatient clinic included anthropometric and anamnestic information, major comorbidities, GOLD stage and the frequency and severity of COPD exacerbations. Respiratory symptoms were assessed as singularly and as part of COPD assessment test (CAT) and modified medical research council (mMRC) dyspnea scale. Patients were categorized according to the GOLD 2025 ABE assessment. Group E included patients with ≥2 moderate exacerbations or ≥1 exacerbation requiring hospitalization in the previous year. Patients with 0 or 1 moderate non-hospitalized exacerbation were classified as Group A when CAT was <10 and mMRC was 0–1, or as Group B when CAT was ≥10 or mMRC was ≥2. As a descriptive sensitivity analysis, GOLD group distribution was recalculated according to the GOLD 2026 criteria [14], which assign patients with ≥1 moderate or severe exacerbation in the previous year to Group E.

As part of their clinical evaluation, all patients performed a flow–volume spirometry and/or body plethysmography (Masterlab Jaeger, Höchberg, Germany) according to ATS/ERS guidelines [15]. Exercise capabilities were assessed with a 6-min walking test (6MWT). Routine blood tests included white blood cell (WBC) count with differential leukocyte count (including BEC), platelets (PLT), serum creatinine, N-terminal pro-B-type natriuretic peptide (NT-proBNP), C-reactive protein (CRP), triglycerides, low-density lipoprotein (LDL) cholesterol, and high-density lipoprotein (HDL) cholesterol. The neutrophil-to-lymphocyte ratio (NLR) was calculated by dividing the absolute neutrophil count by the absolute lymphocyte count. Finally, chest high-resolution computed tomography (HRCT) was performed as part of the routine clinical assessment to characterize structural lung abnormalities and support phenotypic classification. For this retrospective analysis, radiological findings were assessed qualitatively and recorded as binary variables (present/absent), including emphysema, ground-glass opacities, consolidations, interlobular septal thickening, bronchiectasis, mosaic attenuation, centrilobular nodules, and solitary pulmonary nodules. No quantitative CT measurements were performed. Patients without information on exacerbation history or without the assessments required for study inclusion, including blood testing, 6MWT and chest HRCT, were excluded from the final analysis. Sporadic missing values in CAT, mMRC and other retained clustering variables were handled using the imputation procedure described below.

The study was approved by the Bari Institutional Ethics Committees (Ethical Committee number: 1709/CEL) and was conducted according to the Good Clinical Practice standards and the Helsinki Declaration of 1975. All patients signed a written informed consent form before participating in the study.

### 2.2. Statistical Analysis

After assessing distribution with the Shapiro–Wilk test, we considered continuous variables as means ± standard deviations (SD) when normally distributed, while those with non-normal distribution were described using medians and interquartile ranges (IQR). Comparisons were made using Student’s *t*-test or the Mann–Whitney test. Binary variables were expressed as percentages, using Fisher’s exact or the χ^2^ test for comparisons. Missingness was assessed for each candidate clustering variable before analysis. Variables with >20% missing observations were not imputed and were excluded from cluster derivation. For variables retained in the clustering analysis with ≤20% missingness, sporadic missing data were assumed to be missing at random (MAR) and imputed using multiple imputation by chained equations (MICE). Structural missingness was identified in 15 patients who did not undergo the 6MWT and in one patient without an available HRCT assessment; these values were not imputed. In contrast, the 35 missing RV/TLC values were included in the MICE procedure under the MAR assumption. The imputation model included all variables retained for cluster derivation. Five imputed datasets were generated. For each originally missing observation, the five imputed values were averaged, and the resulting single completed dataset was used to calculate the Gower dissimilarity matrix and perform PAM clustering. Variable-specific missingness rates are reported in Supplementary Table S1.

To explore multidimensional patterns within the study population, we conducted an unsupervised cluster analysis based on multidimensional patient-level data. We selected 19 disease-related variables grouped into four domains. The clinical impact domain included CAT score, mMRC score, number of exacerbations in the previous year, exacerbations requiring hospital admission and a 6-min walking distance (6MWD). The lung function domain included FEV_1_% predicted, FVC % predicted, FEV_1_/FVC, and RV/TLC. The radiological domain included emphysema, ground-glass opacities, consolidations, septal thickening, bronchiectasis, mosaic attenuation, centrilobular nodules, and solitary pulmonary nodules. Finally, the systemic inflammation domain included the NLR and CRP. Demographic characteristics, smoking and occupational exposure, comorbidities, GOLD classification, BEC and inhaled treatments were not used to derive the clusters and were subsequently evaluated for cluster characterization (Supplementary Table S2).

Given the combination of quantitative and binary variables, pairwise dissimilarities between patients were calculated using the Gower distance. All 19 variables were assigned equal weights in the primary analysis. Binary variables indicating the presence of clinically or radiologically relevant abnormalities were considered as asymmetric, so that joint absence did not contribute to patient similarity. The resulting dissimilarity matrix was used as input for Partitioning Around Medoids (PAM) clustering, which operates directly on distance matrices and identifies representative observations (medoids) within each cluster. The optimal number of clusters was evaluated by calculating the average silhouette width for solutions ranging from two to eight clusters, with higher values indicating greater within-cluster cohesion and between-cluster separation. The solution with the highest average silhouette width was selected and medoid observations were retained as representative cluster profiles. Cluster stability was assessed using 1000 bootstrap resamples and the agreement between bootstrap-derived and primary cluster assignments was quantified using the adjusted Rand index (ARI) and cluster-specific Jaccard similarity coefficients. The robustness of the primary solution was further examined through sensitivity analyses using alternative binary coding and weighting strategies, leave-one-domain-out analyses and hierarchical clustering with average and complete linkage. For descriptive visualization, principal coordinate analysis (PCoA) was applied to the Gower distance matrix and the first two coordinates were plotted according to cluster membership.

Between-cluster comparisons of variables used for cluster derivation were considered descriptive and were not interpreted as inferential evidence. Inferential testing and Benjamini–Hochberg correction across 34 post hoc comparisons were restricted to variables not used to construct the clusters. GOLD classes were also treated descriptively because they were derived from CAT, mMRC and exacerbation history.

Statistical analyses were performed using IBM SPSS Statistics, version 26 (IBM Corp., Armonk, NY, USA) and R software (version 4.0.2, R Foundation, Kaysville, UT, USA), considering a *p* value < 0.05 as statistically significant.

## 3. Results

### 3.1. Baseline Features

**Figure 1:**
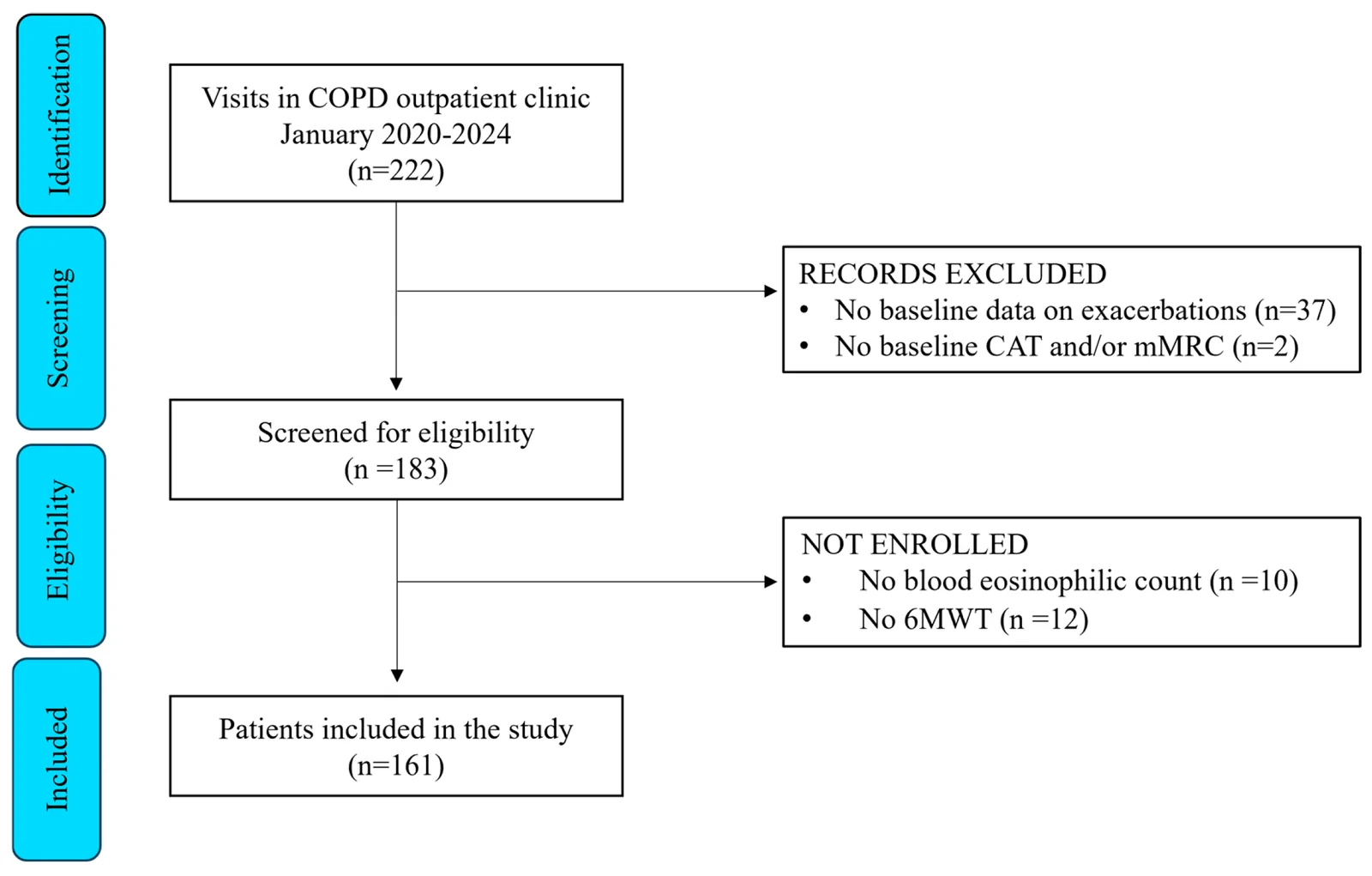
Flow chart showing the enrollment process for the overall population.

After application of the exclusion criteria, 161 patients were included in the final analysis (Figure 1).

The enrolled cohort had a median age of 70 years and was predominantly composed of male patients (77.6%). Most patients were current (39.1%) or former smokers (50.9%), and 31.7% reported a positive history of occupational exposure to respiratory toxic agents (Table 1).

A high burden of cardiovascular comorbidities was observed, with most patients reporting at least one condition, including arterial hypertension (64%), ischemic cardiomyopathy (18.6%), or atrial fibrillation (15.5%). Respiratory symptoms were common, with dyspnea and productive cough being the most frequently reported. This symptom burden was reflected by CAT and mMRC scores, with 45.3% of patients classified as GOLD B. Notably, despite a low rate of hospital admissions due to severe COPD exacerbations (8.1%), up to 27.3% of patients were classified as GOLD E. Applying the GOLD 2026 criteria reclassified 21 patients (13.0%) from Groups A or B to Group E. Consequently, the A/B/E distribution changed from 27.4%/45.3%/27.3% to 24.2%/35.4%/40.4%.

From a radiological standpoint, pulmonary emphysema was observed in 41% of the enrolled patients, whereas bronchiectasis and solitary pulmonary nodules were less frequently detected (16.8% and 16.1%, respectively; Supplementary Table S3).

Finally, blood analyses revealed a median blood eosinophil count of 212 cells/μL, with total WBC counts within the normal range.

### 3.2. Cluster Analysis

**Figure 2:**
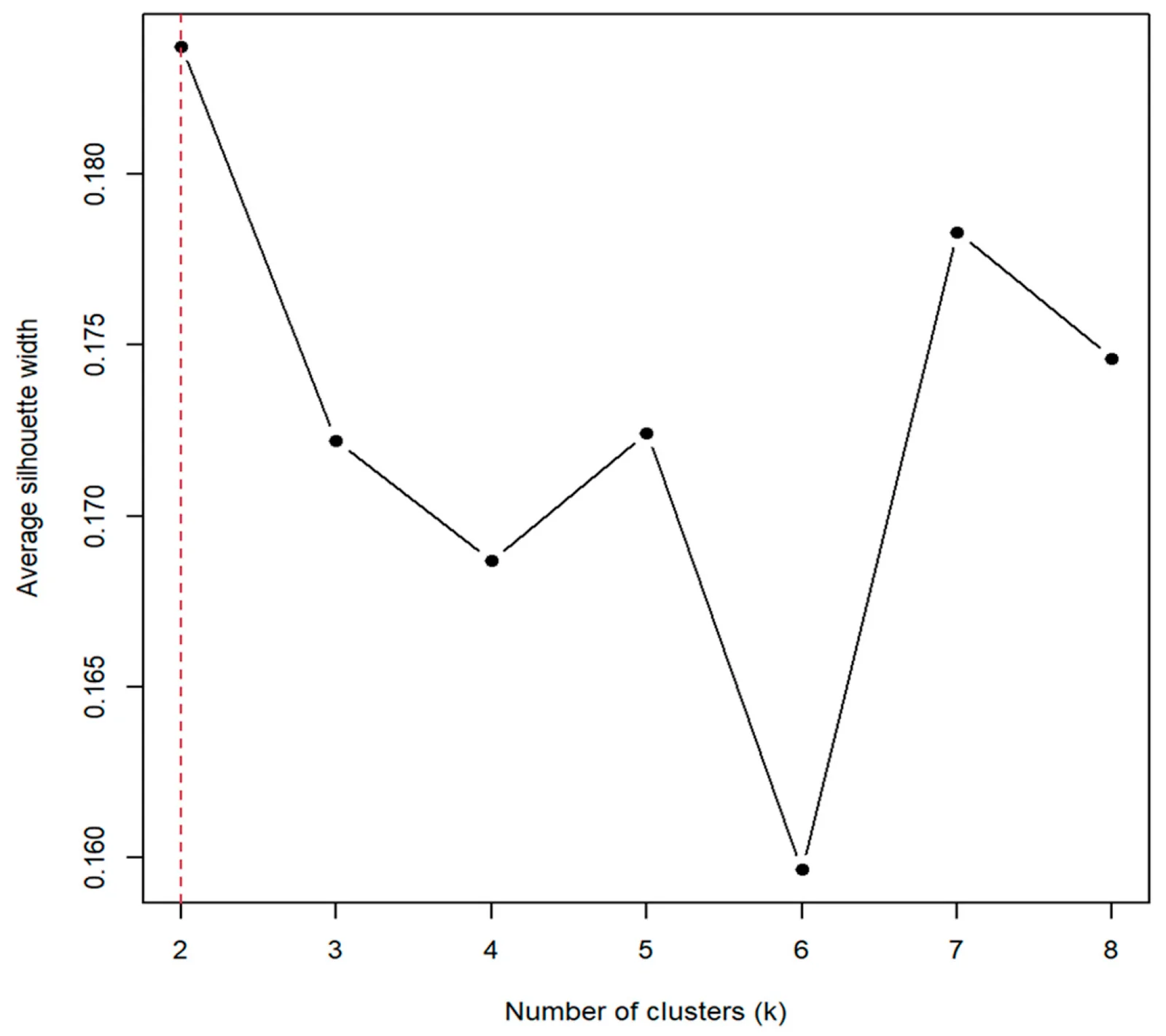
Average silhouette width for clustering solutions ranging from two to eight clusters.

**Figure 3:**
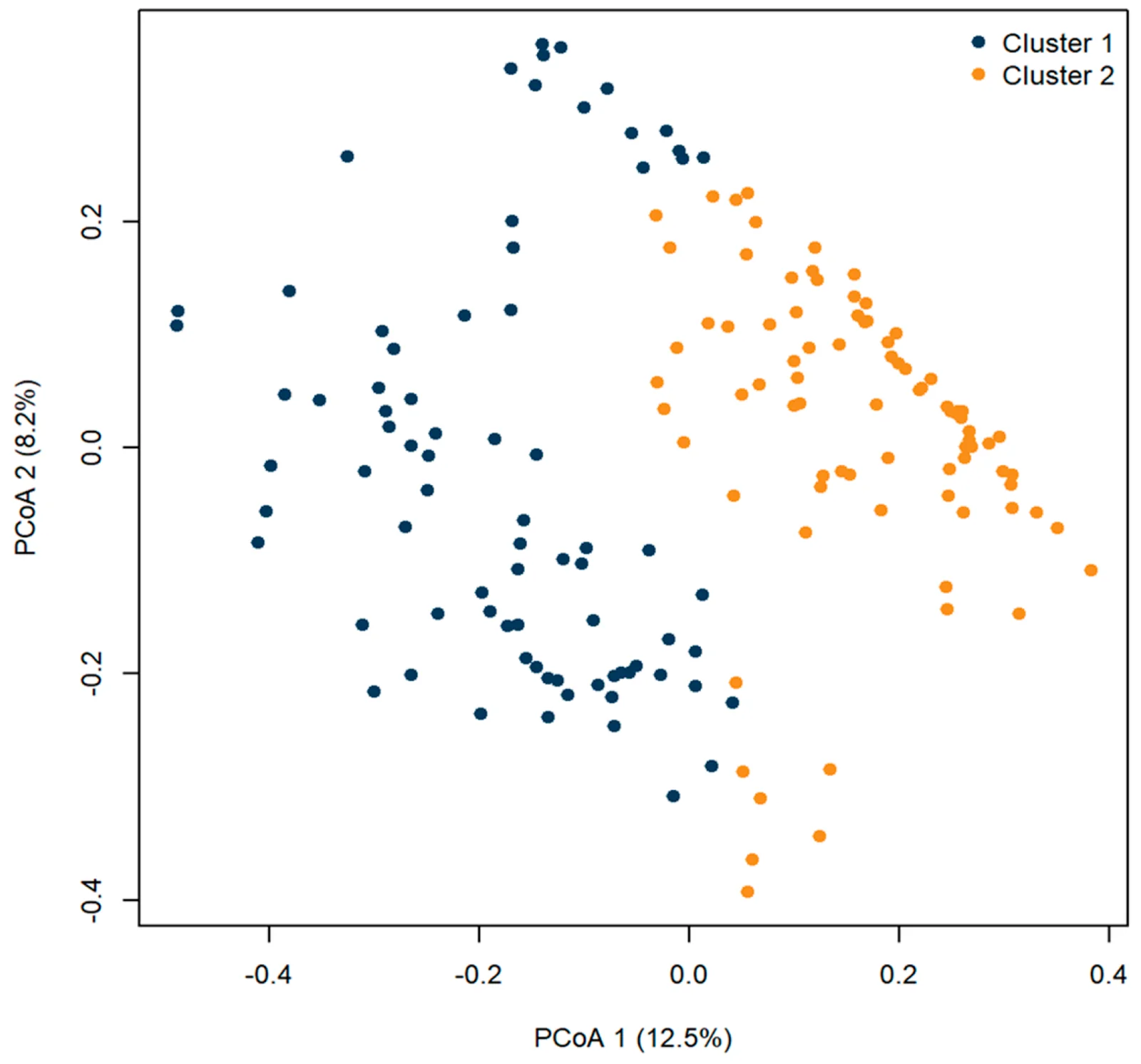
Principal coordinate analysis (PCoA) of the Gower distance matrix according to cluster assignment. Each point represents an individual patient (Cluster 1 in blue; Cluster 2 in orange).

Among the solutions evaluated from k = 2 to k = 8, the two-cluster solution yielded the highest average silhouette width (0.184). However, the absolute value indicated only modest separation between the resulting groups (Figure 2 and Figure 3).

Cluster stability was supported by bootstrap resampling, with a median ARI of 0.951 and median Jaccard similarities of 0.975 and 0.976 for clusters 1 and 2, respectively. A two-cluster solution remained optimal across the prespecified sensitivity analyses, although concordance with the primary partition varied substantially, particularly after exclusion of the radiological domain and with hierarchical clustering (Supplementary Table S4).

No significant differences were observed between clusters in age, sex, BMI, smoking status, atopy, family history of respiratory disease, occupational exposure or overall comorbidity burden (Table 2). Among respiratory symptoms, dyspnea was more frequent in cluster 1 than in cluster 2 (94.9% vs. 83.1%, *p* = 0.023), with no significant differences for cough, phlegm or weight loss.

The variables used to derive the partition are reported descriptively as the dimensions defining the two profiles. Cluster 1 was characterized by a greater exacerbation burden, higher CAT and mMRC scores, shorter 6MWD, lower FEV_1_, FVC and FEV_1_/FVC, and higher RV/TLC. It also showed a higher prevalence of emphysema, ground-glass opacities, septal thickening and solitary pulmonary nodules. Bronchiectasis was numerically more frequent in cluster 1 (23.1% vs. 10.8%). The derived GOLD distribution was also reported descriptively, with GOLD E patients more represented in cluster 1 and GOLD A patients more represented in cluster 2.

Variables used for cluster derivation are reported descriptively because the observed differences are inherent to the clustering procedure. GOLD group is also presented descriptively because it is derived from CAT, mMRC and exacerbation history. Inferential comparisons are reported only for variables not used to derive the clusters.

No significant differences were observed in blood eosinophil counts or in the distribution of eosinophil categories between clusters. Treatment prescriptions, however, differed substantially. Patients in cluster 1 were more frequently treated with LAMA/LABA/ICS triple therapy (66.7% vs. 25.3%, *p* < 0.001), whereas LAMA and ICS/LABA were more common in cluster 2 (*p* = 0.004 and *p* = 0.018, respectively).

After Benjamini–Hochberg correction for multiple post hoc comparisons, only the difference in LAMA/LABA/ICS use remained statistically significant. The differences observed for dyspnea, LAMA and ICS/LABA treatment were no longer significant. No relevant between-cluster differences emerged for demographic characteristics, smoking history, occupational exposure, comorbidity burden, blood eosinophil levels or the other laboratory variables (Supplementary Table S5).

## 4. Discussion

Our study suggests that the integration of clinical, functional, radiological and laboratory features may help characterize different COPD profiles in real-world settings. We previously demonstrated that reliance on COPD classes defined with CAT-mMRC scores could miss several clinically relevant traits, including functional impairment, radiological abnormalities and exposure-related factors that are not adequately captured by symptom-based categorization alone [16]. In the present analysis, the two-cluster solution described two partially overlapping clinical profiles characterized by different distributions of exacerbation burden, respiratory symptoms, functional impairment, radiological abnormalities and treatment patterns. Given the modest silhouette width, these profiles should be interpreted as patterns within a clinical continuum rather than as totally separate COPD phenotypes. Within this continuum, patients assigned to cluster 1 generally showed a more severe clinical profile, with a greater exacerbation burden, higher CAT and mMRC scores, worse lung function, greater static hyperinflation, reduced exercise capacity and more frequent emphysema. The more frequent use of single-inhaler triple therapy (SITT) in cluster 1 was consistent with the greater treatment burden observed in this group, although the cross-sectional design does not allow conclusions regarding the relationship between treatment and disease severity.

Age, sex, smoking status, occupational exposure and overall comorbidity burden were comparable between the two clusters. Interestingly, the total number of comorbidities did not differ significantly between clusters and no differences were observed in the prevalence of cardiovascular diseases. This finding suggests that comorbidity burden was not closely aligned with the differences in clinical severity observed between the two profiles and may independently affect patients’ quality of life.

As reported in several analyses from the ECLIPSE study [2,17,18], comorbidities prevalence does not increase linearly with COPD severity, while different comorbidity clusters could be associated with specific clinical, functional or radiological COPD features. Egerod and colleagues [18] reported three different comorbidity clusters from the simultaneous analysis of the ECLIPSE study and the Groningen severe COPD cohorts: a musculoskeletal, a mental and a circulatory/metabolic cluster. The musculoskeletal cluster, characterized by underweight, sarcopenia and osteoporosis/osteoarthritis, was mainly composed of female patients with a lower fat-free mass index, more emphysema and worse lung function. The mental cluster included patients suffering from depression and anxiety who were characterized by younger women with higher scores on the St. George’s Respiratory Questionnaire (SGRQ). Finally, the circulatory/metabolic cluster included older male patients with increased fat-free mass index values and several cardiovascular/metabolic comorbidities (diabetes, obesity, arterial hypertension, heart failure). Despite this classification, these three clusters did not differ across GOLD clinical classes, confirming that comorbidity burden and patterns are not strictly aligned with symptom burden and exacerbation risk.

BEC was not included among the variables used to derive the clusters and was subsequently evaluated as a potential distinguishing characteristic. However, no significant differences were observed between clusters in either median BEC or the distribution of eosinophil categories. To date, several studies have attempted to clarify the relationship between higher blood eosinophil levels and COPD outcomes, yielding contrasting results. While it is widely recognized that a BEC > 300 cells/μL represents a useful biomarker for a favorable response to ICS [19,20], it remains unclear whether the same threshold can reliably inform COPD prognosis [21]. Kaenmuang et al. reported that patients with stable BEC levels over a one-year follow-up were at higher risk of exacerbations, supporting the relevance of repeated eosinophil measurements over time rather than reliance on single-time assessments [22]. Conversely, studies based on single blood eosinophil determinations have shown that lower BEC values (<150 cells/μL) are associated with an increased risk of hospitalization and mortality [23,24]. In our cohort, however, baseline BEC did not distinguish the more severe clinical profile observed in cluster 1 from the milder profile observed in cluster 2. Within the limits of this cross-sectional analysis, these findings suggest that a single BEC measurement may not adequately capture the broader multidimensional burden of COPD. However, we acknowledge the potential confounding effect of ICS treatment on blood eosinophil levels, as patients in cluster 1 were more frequently treated with SITT compared with those in cluster 2.

From a clinical perspective, these findings suggest that a multidimensional assessment may help recognize patients whose functional or structural disease burden is not fully reflected by symptom- and exacerbation-based classification. This could promote a more comprehensive evaluation of potentially modifiable traits, including exacerbation risk, exercise limitation, static hyperinflation and coexisting radiological abnormalities. However, the identified profiles are not yet ready for direct clinical implementation. External validation, longitudinal assessment of their relationship with clinical outcomes and the development of a simple assignment approach based on routinely available variables would be required before they could be incorporated into clinical pathways.

We must acknowledge some limitations to this study. First, its retrospective and single-center design may limit the generalizability of our findings. The cohort was derived from a tertiary outpatient clinic, potentially introducing referral bias and overrepresentation of more complex cases. Second, although unsupervised clustering offers a data-driven approach without predefined assumptions, the resulting clusters depend on the selected variables, distance metric and algorithm. The modest sample size relative to the 19 variables included in cluster derivation may also have increased susceptibility to sample-specific patterns. Although bootstrap resampling and sensitivity analyses were performed to assess internal stability and robustness, no split-sample or external validation was available; therefore, the reproducibility of the identified clusters in independent COPD populations remains to be established. Third, some variables used to characterize the clusters, including treatment patterns and BEC, were assessed cross-sectionally and may be influenced by treatment exposure and other unmeasured confounders. In particular, blood eosinophils were based on a single baseline measurement, despite their known within-patient variability over time. Repeated longitudinal assessments might provide a more accurate characterization of eosinophilic status and its relationship with clinical outcomes. DLCO was not consistently available and could not be included in the analysis. Its absence limited the functional characterization of the empyema-predominant profile, particularly in cluster 1, and precluded assessment of the relationship between structural abnormalities and gas-transfer impairment. Finally, the relatively low silhouette width indicates that cluster separation was not marked. The identified groups should therefore be interpreted as overlapping clinical profiles rather than completely distinct phenotypes. Further multicenter studies, ideally including external validation, are needed to assess whether these clusters can be reproduced in other COPD populations.

## 5. Conclusions

In conclusion, unsupervised cluster analysis identified two internally stable but partially overlapping multidimensional COPD profiles. These findings suggest that conventional classifications and single biomarkers may not fully capture the complexity of COPD, while a data-driven phenotyping strategy may help improve disease characterization and support a more personalized approach to clinical evaluation and treatment planning.

## Figures and Tables

**Table 1:** Baseline features of the enrolled population.

Patients (n)	161
Age (Years, Median, IQR)	70 [61–76]
Gender (Male/Female, %)	77.6/22.4
BMI (Kg/m^2^, Median, IQR)	27 [24–31]
Smoke habits (%, n)	
Current smoker	39.1 (63)
Former smoker	50.9 (82)
No smoker	9.9 (16)
Atopy (%, n)	18 (29)
Work exposure (%, n)	31.7 (51)
Comorbidities (%, n)	
Arterial hypertension	64 (103)
Ischemic cardiomyopathy	18.6 (30)
Atrial fibrillation	15.5 (25)
Asthma	2.5 (4)
GERD	10.6 (17)
OSAS	15.5 (25)
Type-2 Diabetes	19.9 (32)
Dyslipidemia	33.5 (54)
Thyroid disease	16.1 (26)
Depression	6.8 (11)
Total comorbidities (Median, IQR)	4 [2–5]
Symptoms at first visit (%, n)	
Dyspnea	88.8 (143)
Cough	54.7 (88)
Phlegm	52.2 (84)
Weight loss	1.9 (3)
Exacerbations (Median, IQR)	0 [0–1]
Exacerbations with hospital admission (%, n)	8.1 (13)
GOLD class (%, n)	
GOLD A	27.4 (44)
GOLD B	45.3 (73)
GOLD E	27.3 (44)
CAT ( Median, IQR )	13 [8–17]
mMRC ( Median, IQR )	2 [1–3]
Lung function (Mean, SD)	
FEV_1_ (% predicted)	65.8 ± 19.3
FVC (% predicted)	84 ± 18.3
FEV1/FVC (%)	56.9 ± 11.9
RV/TLC (% predicted)	132.9 ± 26.2
Blood workup	
WBC (cell/mcl)	7480 [6390–8805]
Eosinophils (cell/mcl)	212 [150–295.8]
Creatinine (mg/dL)	0.89 [0.77–1.09]
NT-pro-BNP (pg/mL)	126 [65–269]
CRP (mg/L)	4 [2.9–6.4]
Triglycerides (mg/dL)	111 [82–144]
LDL (mg/dL)	94 [65–129]
HDL (mg/dL)	49 [40–57]

**Table 2:** Clinical, functional, radiological and laboratory features according to cluster classification.

	Cluster 1	Cluster 2	*p* Value
	**(** ***n*** ** = 78)**	**(** ***n*** ** = 83)**	
**Age (years)**	72 [63–77]	69 [60–76]	0.214
**Male/Female (%)**	79.5/20.5	75.9/24.1	0.706
**BMI (kg/m^2^)**	26 [24–31]	27 [24–30.5]	0.881
**Smoke habits (%, ** ***n*** **)**			0.141
Current smoker	41 (32)	37.3 (31)	
Former smoker	53.8 (42)	48.2 (40)	
Never smoker	5.1 (4)	14.5 (12)	
**Atopy (%, ** ***n*** **)**	15.4 (12)	20.5 (17)	0.420
**Familiarity (%, ** ***n*** **)**	23.1 (18)	21.7 (18)	0.852
**Work exposure (%, ** ***n*** **)**	34.6 (27)	28.9 (24)	0.499
**Comorbidities (%, ** ***n*** **)**			
Arterial hypertension	70.5 (55)	57.8 (48)	0.103
Ischemic cardiomyopathy	20.5 (16)	16.9 (14)	0.686
Atrial fibrillation	16.7 (13)	14.5 (12)	0.828
Asthma	2.6 (2)	2.4 (2)	1.000
GERD	9 (7)	12 (10)	0.612
OSAS	16.7 (13)	14.5 (12)	0.828
Type-2 diabetes	20.5 (16)	19.3 (16)	0.847
Dyslipidemia	32.1 (25)	34.9 (29)	0.740
Thyroid disease	14.1 (11)	18.1 (15)	0.527
Depression	5.1 (4)	8.4 (7)	0.537
**Total comorbidities**	4 [2–6]	3 [2–5]	0.244
**Symptoms at first visit (%, ** ***n*** **)**			
Dyspnea	94.9 (74)	83.1 (69)	**0.023**
Cough	57.7 (45)	51.8 (43)	0.527
Phlegm	59 (46)	45.8 (38)	0.115
Weight loss	1.3 (1)	2.4 (2)	1.000
**Exacerbations**	1 [0–2]	0 [0–1]	—
**Exacerbations with hospital admission (%, ** ***n*** **)**	12.8 (10)	3.6 (3)	—
**GOLD class (%, ** ***n*** **)**			—
GOLD A	10.3 (8)	43.4 (36)	
GOLD B	47.4 (37)	43.4 (36)	
GOLD E	42.3 (33)	13.3 (11)	
**CAT**	15 [12–21]	9 [6–13.2]	—
**mMRC**	3 [2–3]	1 [1–2]	—
**6MWD (m)**	312 [142.4–440]	440 [355–500]	—
**Chest CT (%, ** ***n*** **)**			
Emphysema	75.6 (59)	8.4 (7)	—
GGO	15.4 (12)	3.6 (3)	—
Consolidations	9 (7)	8.4 (7)	—
Septal thickening	19.2 (15)	7.2 (6)	—
Bronchiectasis	23.1 (18)	10.8 (9)	—
Mosaic attenuation	1.3 (1)	4.8 (4)	—
Centrilobular nodules	24.4 (19)	12 (10)	—
Solitary nodules	23.1 (18)	9.6 (8)	—
**Lung function**			
FEV_1_ (% predicted)	56.9 ± 17.8	74.1 ± 16.8	—
FVC (% predicted)	78.5 ± 19.2	89.5 ± 15.7	—
FEV_1_/FVC (%)	53.2 [46–61.1]	64 [56.6–67.5]	—
RV/TLC (% predicted)	140 [127.5–160]	124 [114.7–134.1]	—
**Blood workup**			
WBC (cells/µL)	7190 [6430–8710]	7720 [6430–8805]	0.526
Eosinophils (cells/µL)	199 [152–294]	213 [149–294]	0.650
Neutrophil-to-lymphocyte ratio	2.09 [1.6–2.9]	2.04 [1.63–3.08]	—
Eosinophils (%, n)			0.873
≥300 cells/µL	24.4 (19)	24.1 (20)	
150–299 cells/µL	52.6 (41)	49.4 (41)	
<150 cells/µL	23.1 (18)	26.5 (22)	
Creatinine (mg/dL)	0.88 [0.77–1.06]	0.9 [0.78–1.1]	0.997
NT-proBNP (pg/mL)	122 [68–269]	130 [55–262]	0.949
CRP (mg/L)	4 [2.9–6.3]	4 [4–6.4]	—
Triglycerides (mg/dL)	104 [80–135]	115 [88–156]	0.120
LDL (mg/dL)	92 [67–125]	96 [66–133]	0.664
HDL (mg/dL)	51 [42–56]	47 [38–58]	0.242
**Treatments (%, ** ***n*** **)**			
LAMA	6.4 (5)	22.9 (19)	**0.004**
LAMA/LABA	17.9 (14)	31.3 (26)	0.068
LAMA/LABA/ICS	66.7 (52)	25.3 (21)	**<0.001**
ICS/LABA	2.6 (2)	13.3 (11)	**0.018**

## Data Availability

The data that support the findings of this study are available from the corresponding author upon reasonable request.

## Supplementary Materials

The following supporting information can be downloaded at: https://www.mdpi.com/article/10.3390/medicina62091656/s1, Table S1. Missingness rates in variables assessed before multiple imputation; Table S2. Variables considered for cluster analysis: inclusion, Gower coding, weighting, and reasons for exclusion; Table S3. Radiological features and treatment of the overall cohort; Table S4. Internal stability and sensitivity analyses of the primary two-cluster solution; Table S5. Clinical, functional, radiological and laboratory characteristics according to cluster classification, with adjusted *p* values after post hoc comparisons.

## Abbreviations

The following abbreviations are used in this manuscript:

ARI	Adjusted Rand index
ATS	American Thoracic Society
BEC	Blood eosinophil count
BMI	Body mass index
CAT	COPD Assessment Test
COPD	Chronic obstructive pulmonary disease
CRP	C-reactive protein
CT	Computed tomography
ERS	European Respiratory Society
FEV1	Forced expiratory volume in 1 s
FVC	Forced vital capacity
GERD	Gastroesophageal reflux disease
GGO	Ground-glass opacity
GOLD	Global Initiative for Chronic Obstructive Lung Disease
HDL	High-density lipoprotein cholesterol
HRCT	High-resolution computed tomography
ICS	Inhaled corticosteroids
IQR	Interquartile range
LABA	Long-acting β2-agonist
LAMA	Long-acting muscarinic antagonist
LDL	Low-density lipoprotein cholesterol
MAR	Missing at random
MICE	Multiple imputation by chained equations
mMRC	Modified Medical Research Council dyspnea scale
NLR	Neutrophil-to-lymphocyte ratio
NT-pro-BNP	N-terminal pro–B-type natriuretic peptide
OSAS	Obstructive sleep apnea syndrome
PAM	Partitioning Around Medoids
PCoA	Principal coordinate analysis
PLT	Platelets
RV/TLC	Residual volume-to-total lung capacity ratio
SD	Standard deviation
SITT	Single-inhaler triple therapy
WBC	White blood cells
6MWD	Six-minute walking distance
6MWT	Six-minute walking test
