# Supplementary material for: Exploratory Identification of Multidimensional COPD Clusters Using Unsupervised Analysis
Source: Medicina (Kaunas). 2026 Aug 29;62(9):1656. doi: 10.3390/medicina62091656 (PMC13608815; doi:10.3390/medicina62091656)
Supplement: Supplementary file 1 [file medicina-62-01656-s001.zip › medicina-4470014-supplementary.pdf]

Article

Exploratory Identification of Multidimensional COPD Clusters Using Unsupervised Analysis

1. Supplemental material

Table S1. Missingness rates in variables assessed before multiple imputation

Variable	Missing values, n	Missingness, %
Background characteristics		
Age	0	0.0
Sex	0	0.0
BMI	11	5.0
Smoking status	0	0.0
Atopy	0	0.0
Family history of respiratory disease	0	0.0
Occupational exposure	0	0.0
Comorbidities		
Arterial hypertension	0	0.0
Ischemic cardiomyopathy	0	0.0
Atrial fibrillation	0	0.0
Asthma	0	0.0
Gastroesophageal reflux disease	0	0.0
Obstructive sleep apnea syndrome	0	0.0
Cancer	0	0.0
Chronic kidney disease	0	0.0
Type 2 diabetes	0	0.0

Variable	Missing values, n	Missingness, %
Dyslipidemia	0	0.0
Thyroid disease	0	0.0
Depression	0	0.0
Total number of comorbidities	0	0.0
Respiratory symptoms		
Dyspnea	0	0.0
Cough	0	0.0
Phlegm	0	0.0
Weight loss	0	0.0
Clinical impact and classification		
Exacerbations in the previous year	0	0.0
Exacerbations requiring hospital admission	0	0.0
GOLD group (derived)	0	0.0
CAT score	24	10.8
mMRC grade	15	6.8
6-minute walk distance (m)	43	19.4
Lung function and exercise-related assessments		
FEV_1 (% predicted)	17	7.7
FVC (% predicted)	20	9.0
FEV_1/FVC (%)	18	8.1
RV/TLC (% predicted)	35	15.8
DLCO (% predicted)	91	41.0
Borg score, pre-6MWT	113	50.9
Borg score, post-6MWT	113	50.9
Heart rate, pre-6MWT	109	49.1
Heart rate, post-6MWT	109	49.1
SpO_2, pre-6MWT	81	36.5

Variable	Missing values, n	Missingness, %
SpO_2, post-6MWT	83	37.4
HRCT findings		
Emphysema	1	0.5
Ground-glass opacities	1	0.5
Consolidations	1	0.5
Interlobular septal thickening	1	0.5
Bronchiectasis	1	0.5
Mosaic attenuation	1	0.5
Centrilobular nodules	1	0.5
Solitary pulmonary nodules	1	0.5
Laboratory variables		
White blood cell count	0	0.0
Blood eosinophil count	0	0.0
Blood eosinophil category	0	0.0
Neutrophil-to-lymphocyte ratio	0	0.0
Creatinine	4	1.8
NT-proBNP	12	5.4
C-reactive protein	8	3.6
Triglycerides	2	0.9
LDL cholesterol	6	2.7
HDL cholesterol	4	1.8
Treatments		
LAMA	0	0.0
LAMA/LABA	0	0.0
LAMA/LABA/ICS (SITT)	0	0.0
ICS/LABA	0	0.0

Missingness was calculated using N = 222 as the denominator for every raw variable. Category levels (e.g., smoking status, GOLD groups, and eosinophil categories) were not repeated as separate variables. GOLD group was a derived classification and was complete in the derived analysis dataset; missingness of its underlying source scores is reported separately for CAT and mMRC. For 6MWT-related measures, the totals include 15 patients who did not undergo the assessment; these structural missing values were not imputed. The 43 missing 6MWD values comprised 15 structural and 28 sporadic missing values and only the latter were imputed. Borg scores, heart rate, DLCO and SpO_2 had >20% missingness, so they were not imputed and were excluded from cluster derivation. The 35 missing RV/TLC values were included in the MICE procedure. The single missing value for each HRCT feature corresponded to the same patient without an available HRCT assessment and was not imputed.

Table S2. Variables considered for cluster analysis: inclusion, Gower coding, weighting, and reasons for exclusion.

Domain	Variables	Gower coding and weight / reason for exclusion
Included in cluster derivation (primary analysis)		
**Clinical impact**	CAT; mMRC; exacerbations in the previous year; 6MWD	Quantitative; range-standardized by Gower; primary weight 1 each.
**Clinical impact**	Exacerbation requiring hospital admission	Asymmetric binary; primary weight 1.
**Lung function**	FEV_1, % predicted; FVC, % predicted; FEV_1/FVC; RV/TLC, % predicted	Quantitative; range-standardized by Gower; primary weight 1 each.
**Radiology**	Emphysema; ground-glass opacities; consolidations; septal thickening; bronchiectasis; mosaic attenuation; centrilobular nodules; solitary pulmonary nodules	Asymmetric binary; primary weight 1 each.
**Systemic inflammation**	Neutrophil-to-lymphocyte ratio; CRP	Quantitative; range-standardized by Gower; primary weight 1 each.
Excluded from cluster derivation		
**Background characteristics**	Age; sex; BMI; smoking status; atopy; family history; occupational exposure	Not used to derive clusters because they describe background or risk factors rather than current COPD manifestations; evaluated post hoc.
**Comorbidities**	Individual comorbidities; total number of comorbidities	Excluded to focus cluster derivation on COPD-related manifestations; evaluated post hoc.
**Individual symptoms**	Dyspnea; cough; phlegm; weight loss	Excluded to limit redundancy with CAT and mMRC; evaluated post hoc.

Domain	Variables	Gower coding and weight / reason for exclusion
**Derived classification**	GOLD group	Excluded to avoid redundancy with symptoms and exacerbation history already included; reported descriptively only.
**Biomarker**	Blood eosinophil count	Excluded a priori so that its distribution could be assessed independently after cluster derivation.
**Treatments**	LAMA; LAMA/LABA; LAMA/LABA/ICS; ICS/LABA	Excluded because treatments reflect prior prescribing decisions and could introduce circularity; evaluated post hoc.
**Other laboratory variables**	WBC; creatinine; NT-proBNP; triglycerides; LDL; HDL	Not included in the predefined systemic inflammation domain; evaluated post hoc.
**High-missingness assessments**	Borg score and heart rate before/after 6MWT; DLCO; SpO_2 before/after 6MWT	>20% missingness; not imputed and excluded according to the prespecified threshold.

Table S3. Radiological features and treatment of the overall cohort.

Patients (n)	161
**HRCT findings (%, n)**	
Emphysema	41 (66)
Ground-glass opacities	9.3 (15)
Consolidations	8.7 (14)
Septal thickening	13 (21)
Bronchiectasis	16.8 (27)
Mosaic attenuation	3.1 (5)
Centrilobular nodules	18 (29)
Solitary nodules	16.1 (26)

Treatments (%, n)	
LAMA	14.9 (24)
LAMA/LABA	24.8 (40)
SITT	45.3 (73)
ICS/LABA	8.1 (13)

Table S4. Internal stability and sensitivity analyses of the primary two-cluster solution.

Analysis	Best k	Silhouette (k = 2)	ARI vs primary	Cluster sizes (n)	Stability summary (median; 2.5th–97.5th percentile)
**Primary analysis (Gower distance + PAM)**	2	0.184	Reference	78 / 83	—
Bootstrap stability — ARI	2 (fixed)	—	—	—	0.951 (0.344–1.000)
Bootstrap stability — Jaccard, Cluster 1	2 (fixed)	—	—	—	0.975 (0.597–1.000)

Bootstrap stability — Jaccard, Cluster 2	2 (fixed)	—	—	—	0.976 (0.705–1.000)
**Sensitivity analyses**					
Symmetric binary coding	2	0.201	0.810	70 / 91	—
Domain-balanced weighting	2	0.177	0.722	68 / 93	—
Exclusion of clinical impact domain	2	0.273	0.489	68 / 93	—
Exclusion of lung function domain	2	0.229	0.620	77 / 84	—
Exclusion of radiological domain	2	0.278	0.249	60 / 101	—
Exclusion of systemic inflammation domain	2	0.195	1.000	78 / 83	—
Hierarchical clustering, average linkage	2	0.295	0.002	159 / 2	—
Hierarchical clustering, complete linkage	2	0.129	0.120	70 / 91	—

ARI, adjusted Rand index; PAM, Partitioning Around Medoids. The primary analysis used Gower distance, equal weighting of the 19 clustering variables, asymmetric coding of binary clinical/radiological features and PAM. For sensitivity analyses, average silhouette width and cluster sizes refer to the two-cluster solution. ARI quantifies agreement in individual cluster assignments with the primary solution, with values closer to 1 indicating greater concordance. Bootstrap stability was assessed across 1000 valid replications.

Table S5. Clinical, functional, radiological and laboratory characteristics according to cluster classification, with adjusted P values after post hoc comparisons.

	**Cluster 1 (n = 78)**	**Cluster 2 (n = 83)**	**Adjusted P value**
**Age (years)**	72 [63–77]	69 [60–76]	0.677

	Cluster 1 (n = 78)	Cluster 2 (n = 83)	Adjusted P value
**Male/Female (%)**	79.5/20.5	75.9/24.1	0.992
**BMI (kg/m²)**	26 [24–31]	27 [24–30.5]	0.992
**Smoke habits (%, n)**			0.509
Current smoker	41 (32)	37.3 (31)	
Former smoker	53.8 (42)	48.2 (40)	
Never smoker	5.1 (4)	14.5 (12)	
**Atopy (%, n)**	15.4 (12)	20.5 (17)	0.992
**Familiarity (%, n)**	23.1 (18)	21.7 (18)	0.992
**Work exposure (%, n)**	34.6 (27)	28.9 (24)	0.992
**Comorbidities (%, n)**			
Arterial hypertension	70.5 (55)	57.8 (48)	0.480
Ischemic cardiomyopathy	20.5 (16)	16.9 (14)	0.992
Atrial fibrillation	16.7 (13)	14.5 (12)	0.992
Asthma	2.6 (2)	2.4 (2)	1.000
GERD	9 (7)	12 (10)	0.992
OSAS	16.7 (13)	14.5 (12)	0.992
Type-2 diabetes	20.5 (16)	19.3 (16)	0.992
Dyslipidemia	32.1 (25)	34.9 (29)	0.992
Thyroid disease	14.1 (11)	18.1 (15)	0.992
Depression	5.1 (4)	8.4 (7)	0.992
**Total comorbidities**	4 [2–6]	3 [2–5]	0.677

	Cluster 1 (n = 78)	Cluster 2 (n = 83)	Adjusted P value
**Symptoms at first visit (%, n)**			
Dyspnea	94.9 (74)	83.1 (69)	0.211
Cough	57.7 (45)	51.8 (43)	0.992
Phlegm	59 (46)	45.8 (38)	0.480
Weight loss	1.3 (1)	2.4 (2)	1.000
**Exacerbations**	1 [0–2]	0 [0–1]	—
**Exacerbations with hospital admission (%, n)**	12.8 (10)	3.6 (3)	—
**GOLD class (%, n)**			—
GOLD A	10.3 (8)	43.4 (36)	
GOLD B	47.4 (37)	43.4 (36)	
GOLD E	42.3 (33)	13.3 (11)	
**CAT**	15 [12–21]	9 [6–13.2]	—
**mMRC**	3 [2–3]	1 [1–2]	—
**6MWD (m)**	312 [142.4–440]	440 [355–500]	—
**Chest CT (%, n)**			
Emphysema	75.6 (59)	8.4 (7)	—
GGO	15.4 (12)	3.6 (3)	—
Consolidations	9 (7)	8.4 (7)	—
Septal thickening	19.2 (15)	7.2 (6)	—
Bronchiectasis	23.1 (18)	10.8 (9)	—
Mosaic attenuation	1.3 (1)	4.8 (4)	—

	Cluster 1 (n = 78)	Cluster 2 (n = 83)	Adjusted P value
Centrilobular nodules	24.4 (19)	12 (10)	—
Solitary nodules	23.1 (18)	9.6 (8)	—
**Lung function (% predicted)**			
FEV_1	56.9 ± 17.8	74.1 ± 16.8	—
FVC	78.5 ± 19.2	89.5 ± 15.7	—
FEV_1/FVC	53.2 [46–61.1]	64 [56.6–67.5]	—
RV/TLC	140 [127.5–160]	124 [114.7–134.1]	—
**Blood workup**			
WBC (cells/μL)	7190 [6430–8710]	7720 [6430–8805]	0.992
Eosinophils (cells/μL)	199 [152–294]	213 [149–294]	0.992
Neutrophil-to-lymphocyte ratio†	2.09 [1.6–2.9]	2.04 [1.63–3.08]	—
Eosinophils (%, n)			0.992
≥300 cells/μL	24.4 (19)	24.1 (20)	
150–299 cells/μL	52.6 (41)	49.4 (41)	
<150 cells/μL	23.1 (18)	26.5 (22)	
Creatinine (mg/dL)	0.88 [0.77–1.06]	0.9 [0.78–1.1]	1.000
NT-proBNP (pg/mL)	122 [68–269]	130 [55–262]	1.000
CRP (mg/L)	4 [2.9–6.3]	4 [4–6.4]	—
Triglycerides (mg/dL)	104 [80–135]	115 [88–156]	0.480
LDL (mg/dL)	92 [67–125]	96 [66–133]	0.992
HDL (mg/dL)	51 [42–56]	47 [38–58]	0.677

	**Cluster 1 (n = 78)**	**Cluster 2 (n = 83)**	**Adjusted P value**
**Treatments (%, n)**			
LAMA	6.4 (5)	22.9 (19)	0.067
LAMA/LABA	17.9 (14)	31.3 (26)	0.405
LAMA/LABA/ICS	66.7 (52)	25.3 (21)	**<0.001**
ICS/LABA	2.6 (2)	13.3 (11)	0.211
